# Association of Group B *Streptococcus* Colonization and Bovine Exposure: A Prospective Cross-Sectional Cohort Study

**DOI:** 10.1371/journal.pone.0008795

**Published:** 2010-01-20

**Authors:** Shannon D. Manning, A. Cody Springman, Amber D. Million, Nicole R. Milton, Sara E. McNamara, Patricia A. Somsel, Paul Bartlett, H. Dele Davies

**Affiliations:** 1 Microbial Evolution Laboratory, Michigan State University, East Lansing, Michigan, United States of America; 2 National Food Safety and Toxicology Center, Michigan State University, East Lansing, Michigan, United States of America; 3 Department of Pediatrics and Human Development, Michigan State University, East Lansing, Michigan, United States of America; 4 Bureau of Laboratories, Michigan Department of Community Health, Lansing, Michigan, United States of America; Columbia University, United States of America

## Abstract

**Background:**

While Group B *Streptococcus* (GBS) human colonization and infection has long been suspected as originating from cows, several investigators have suggested that ongoing interspecies GBS transmission is unlikely due to genotyping data demonstrating that human and bovine-derived GBS strains represent mostly distinct populations. The possibility of ongoing transmission between humans and their livestock has not been systematically examined.

**Methodology/Principal Findings:**

To examine ongoing interspecies transmission, we conducted a prospective cross-sectional cohort study of 68 families and their livestock. Stool specimens were collected from 154 people and 115 livestock; GBS was detected in 19 (12.3%) humans and 2 (1.7%) animals (bovine and sheep). Application of multilocus sequence typing (MLST) identified 8 sequence types (STs or clones), with STs 1 and 23 predominating. There were 11 families in which two members submitted stools and at least one had GBS colonization. In 3 of these families, both members (consisting of couples) were colonized, yielding a co-colonization rate of 27% (95% CI: 7%–61%). Two of these couples had strains with identical MLST, capsule (*cps*) genotype, susceptibility, and RAPD profiles. One couple co-colonized with ST-1 (*cps*5) strains also had a bovine colonized with the identical strain type. On multivariate analysis of questionnaire data, cattle exposure was a predictor of GBS colonization, with each unit increase in days of cattle exposure increasing the odds of colonization by 20% (*P* = 0.02). These results support interspecies transmission with additional evidence for transmission provided by the epidemiological association with cattle exposure.

**Conclusions/Significance:**

Although GBS uncommonly colonizes livestock stools, increased frequency of cattle exposure was significantly associated with human colonization and one couple shared the same GBS strains as their bovine suggesting intraspecies transmission. These results set the framework for GBS as a possible zoonotic infection, which has significant public health implications.

## Introduction

Group B *Streptococcus* (GBS) frequently causes neonatal sepsis and meningitis as a result of transmission from mothers to neonates during childbirth. During pregnancy, up to 36% [1] of women are colonized with GBS, as are ∼45% of neonates born to colonized mothers [2]. Asymptomatic GBS colonization also occurs frequently in young adults (20–40%) [3], [4] and the elderly (22%) [5]. Little is known about the likelihood of GBS transmission between animals and humans. Based on the sudden emergence of GBS in neonatal disease in the 1960's after a long history of contributing mostly to bovine infections [6], it was postulated that GBS originated from bovines. However, it has subsequently been suggested by several investigators that interspecies GBS transmission is not likely because genotyping data has demonstrated that human- and bovine-derived GBS strains represent mostly distinct populations [7], [8], [9], [10], [11], [12], [13]. Because the fecal-oral transmission route has been shown to be important for colonization in various human populations [14], it is plausible that direct contact with feces from colonized animals can contribute to human colonization. Indeed, GBS has been isolated from other domesticated animals including dogs, cats, rabbits, horses, guinea pigs [15] and goats [10], but no environmental reservoirs have been identified.

Although the frequency of asymptomatic bovine colonization is not known, GBS commonly causes mastitis [16]. Nevertheless, epidemiologic linkage studies have not been conducted to examine transmission between humans and asymptomatically colonized bovines. Here, we determined whether individuals who come in regular contact with bovines are more frequently colonized, and if there is evidence for interspecies transmission.

## Results

### Population Characteristics

This GBS prevalence study involved 269 of 361 (74.5%) stools collected from 107 of 115 families (154 of the 180 persons) participating in a larger study of enteric pathogens. In the subset, 101 people representing 68 households provided a self collected stool and at least one stool from their livestock. Eighteen of the 154 individuals submitted two livestock samples while 53 people only provided a stool sample from themselves.

Among the 269 stools, over half (*n* = 154, 57.2%) were from people and 115 were from cattle (*n* = 50, 18.6%), sheep (*n* = 39, 14.5%), pigs (*n* = 13, 4.8%), horses (*n* = 10, 3.7%), or goats (*n* = 3, 1.1%). Matched human-livestock stools were available for 101 of the 154 (65.6%) people. Matched human-human stools from two people in the same household were available for 112 people (72.7%) including 17 sets of siblings, 13 couples, and 26 children and parent/guardian pairs.

In all, 65 (42.5%) participants were children between 9 and 17 years of age, 21 (13.7%) were young adults between 18 and 24 years, and 66 (43.1%) were parents or guardians between 27 and 83 years. There were 87 female and 66 male participants; the age of one person was not known.

### Colonization and Molecular Characteristics

GBS was detected in 21 of 269 (7.8%) stools; 19 of 154 (12.3%) strains were from people and 2 of 115 (1.7%) were from animals, namely a cow and sheep. The GBS prevalence among cattle and sheep was 4.0% (95% CI: 0.7%–15%) and 2.6% (95% CI: 0.4%–13%), respectively. All 19 colonized participants were over 18 years of age yielding a colonization rate of 21.8% (95% CI: 14%–32%) in this age group. Twelve (63.2%) of the colonized participants were women, 11 (57.9%) were from different families in which two household members participated, and 15 (78.9%) submitted matching stool specimens from at least one animal including 11 from bovines.

**Figure 1:**
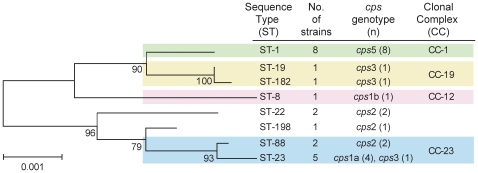
Neighbor joining phylogeny of 21 group B *Streptococcus* (GBS) strains. Phylogeny is based on concatenated multilocus sequence typing data for seven housekeeping genes and the capsule (*cps*) genotypes are provided.

Application of MLST identified eight STs among the 21 GBS strains, with STs 1 and 23 predominating followed by STs 88 and 22 (Figure 1). All eight STs, which have been identified previously[17], represent four clonal complexes (CCs) in the neighbor-joining phylogeny (Figure 1). The complexes were established in a prior unrelated study[18], and represent those STs that grouped together with more than 85% bootstrap support. The bovine and sheep strains were characterized as ST-1 (*cps*V) and ST-23 (*cps*1a), respectively. Overall, the most prevalent *cps* genotypes were *cps*5 (*n* = 8; 38.0%) and *cps*1a (*n* = 5; 23.8%), which were found among the two predominant STs (Figure 1). Five of the 19 (26%) human GBS strains were resistant to erythromycin, clindamycin and azithromycin as was the bovine strain. Moreover, five of the six resistant strains were categorized as ST-1 and one was ST-22.

### Co-Colonization Frequencies

**Figure 2:**
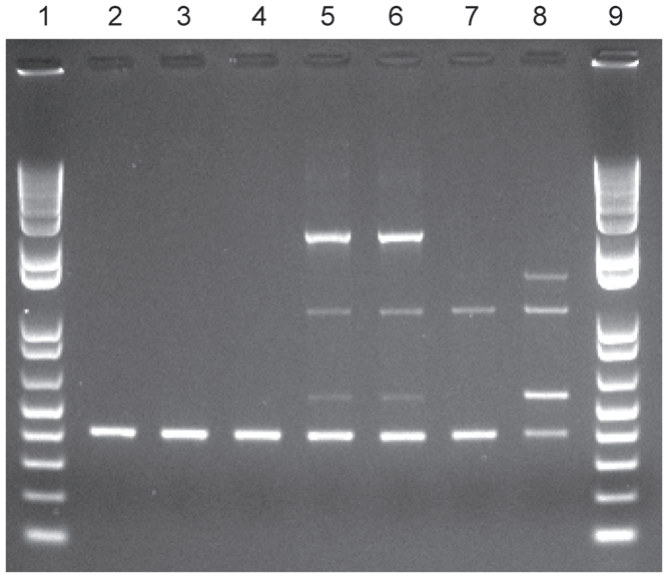
RAPD patterns of GBS strains from epidemiologically linked co-colonized participants. The OPB17 primers, previously described by Martinez et al.^9^ and two additional primer sets^9^ demonstrated similar banding patterns between the married couple and their bovine (Lanes 2–4) and the other co-colonized couple (Lanes 5–6). Lanes 7 and 8 represent additional strains isolated from two unrelated participants, and lanes 1 and 9 are a 1kb plus ladder.

Among the 11 families in which two members submitted stools, GBS was detected in both members from three families, yielding an overall co-colonization frequency of 27% (95% CI: 7%–61%). All three co-colonized families were married couples and two of the three couples had GBS strains with identical MLST, *cps*, susceptibility, and RAPD profiles (Figure 2). One couple was co-colonized with ST-88 (*cps*2) GBS strains, whereas another couple had ST-1 (*cps*5) strains. The third co-colonized couple had two distinct strain types (ST-1, *cps5* and ST-19, *cps3*), yielding a lower (18.2%; 95% CI: 3%–66%) frequency of co-colonization with the identical strain type. Interestingly, the couple co-colonized with ST-1 (*cps*5) strains also had a bovine colonized with the identical strain type; all three strains also were resistant to erythromycin, azithromycin and clindamycin (Figure 2). The stools from the animals raised by all other families in which one person had GBS, were culture negative as were both family members of the colonized sheep.

### Predictors of Colonization

Overall, 11 of the 50 (22.0%; 95% CI: 12%–36%) individuals who submitted a bovine specimen were colonized, a proportion that was not significantly different than the four of 51 (7.8%; 95% CI: 3%–20%) colonized individuals who submitted a specimen from another animal species (Table 1). However, analysis of the survey data demonstrated that participants exposed to cattle in the past week had higher rates of GBS relative to those without cattle exposure (Table 1). The association with cattle exposure was not correlated with colonization by specific STs or *cps* genotypes. In addition, a significant trend (Mantel-Haenszel X^2^: 5.1; degree of freedom (df): 1; *P* = 0.02) was observed between increasing GBS colonization frequencies and higher levels of cattle exposure. No associations were identified between colonization and exposure to other animals, but a significant crude association with GBS colonization was observed for age over 18 years (Table 2). The multivariate analyses confirmed that both cattle exposure and older age were predictors of GBS colonization in this population (Table 2). Specifically, each unit increase in days of cattle exposure increased the odds of GBS colonization by 20%, while participants over 18 were nearly 12 times more likely to be colonized than younger participants.

**Table 1:** Unadjusted associations between group B *Streptococcus* (GBS) colonization and participant characteristics.

Characteristics^a^	Total N	No. Colonized	% Colonized	X^2^	df	Univariate OR	(95% CI)	*P*
**Age**								
≤18 years	77	2	2.6			1.0	–	–
18–40 years	37	9	24.3	15.6	2	12.1	2.21, 118.42	0.0002
≥41 years	39	8	20.5			9.7	1.76, 96.50	0.001
**Gender**								
Male	66	7	10.6					
Female	86	12	14.0	0.4	1	1.0	0.55, 1.62	–
**Primary type of animal** b								
Other	51	4	7.8			1.0		
Cattle	50	11	22.0	6.0	2	3.3	0.88, 15.24	
None	53	4	7.8			1.0	0.18, 5.94	
**Level of cattle exposure in past week**								
None	80	6	7.5			1.0		
1–4 times	17	2	11.8	6.2	2	1.6	0.15, 10.37	0.56
5–7 times	56	11	19.6			3.0	0.94, 10.96	0.04
**Level of pig exposure in past week**								
None	78	7	9.0			1.0		
1–4 times	12	2	16.7	1.8	2	2.0	0.18, 12.81	0.41
5–7 times	63	10	15.9			1.9	0.62, 6.03	0.21
**Level of sheep exposure in past week**								
None	94	14	14.9			1.0		
1–4 times	4	1	25.0	2.5	2	1.9	0.03, 25.55	0.58
5–7 times	55	4	7.3			0.5	0.10, 1.54	0.17
**Level of horse exposure in past week**								
None	95	14	14.7			1.0		
1–4 times	13	3	23.1	5.0	2	1.7	0.27, 7.92	0.44
5–7 times	45	2	4.4			0.3	0.03, 1.27	0.07

Note: The Likelihood Ratio Chi square (X^2^, degree of freedom (df)) was used to examine associations, which are represented by odds ratios (OR), 95% confidence intervals (95% CI), and P values (*P*). Fisher's exact test was used for variables with cell values <5.

a Not all numbers add up to 154 because of missing data in some categories.

b Other category includes pigs, sheep and/or horses; “None” refers to those individuals who failed to submit an animal specimen.

**Table 2:** Predictors of group B *Streptococcus* (GBS) colonization as determined by multivariate analyses.

Characteristics	Multivariate Analysis^b^ OR	95% CI	*P*
**Age**			
≤18 years	1.0		–
18–40 years	11.97	2.294, 62.508	0.003
≥41 years	14.39	2.655, 77.939	0.002
**Gender**			
Male	1.0		–
Female	1.66	0.519, 5.311	0.39
**Cattle exposure in past week**	1.20	1.002, 1.447	0.05
**Pig exposure in past week**	1.07	0.845, 1.200	0.94
**Sheep exposure in past week**	0.99	0.804, 1.208	0.89
**Horse exposure in past week**	0.85	0.696, 1.047	0.13

Note: Odds ratios (OR), 95% confidence intervals (95% CI), and P values (*P*) were estimated using multinomial logistic regression while adjusting for all variables in the table (age, gender and days of exposure in the past week to cattle, sheep, pigs, and horses). Animal exposure variables were included as continuous variables and represent the number of times in the past week in which an individual was near a given animal species.

## Discussion

In this study that involved sampling stools from livestock and their caretakers, we identified probable linkages between cattle exposure and GBS colonization in humans. The fecal GBS colonization rate (21.3%) in the enrolled population of healthy individuals over 18 years of age was similar to rates in other studies using rectal swabs [3], [4], [5], [19]. The finding that adults were more frequently colonized than children is also consistent with previous reports [20], [21], [22], [23]. While the colonization rate (∼2%) in the sampled cohort of livestock was much lower, it is similar to the rate (2.6%) from bulk milk samples of dairy herds [24], [25], [26]. Nonetheless, we suspect that it represents an underestimate, as the GBS isolation rate has been found to be higher in rectal versus fecal specimens at least in human populations [21], [22]. The prevalence of bovine rectal colonization is not well studied, as most studies have examined milk from symptomatic herds [16].

It is not surprising that two of 11 co-colonized married couples shared identical strains since sexual contact is important for colonization [14]. More significantly, one couple had the same strain types with identical susceptibility profiles as their bovine, which is suggestive of interspecies transmission. This is consistent with two prior reports that commented on the possibility of transmission between bovines with mastitis and humans. These two studies, however, did not screen asymptomatic cows, used less sensitive molecular methods leaving doubts about strain relatedness [27], or had limited sampling schemes [28]. For example [28], a farm worker had the identical GBS isolate in his tonsil as in herd milk samples, although no information was given as to whether the worker drank unpasteurized milk from the herd. We hypothesize that bovine mastitis is correlated with higher bacterial colony counts, which may be associated with a greater transmission risk. Similarly, a higher level of contact with a colonized animal with or without mastitis is also likely to be important; this hypothesis is supported by our observation that GBS colonization was associated with an increasing frequency of cattle exposure. Nevertheless, it is important to note that our study population is unique in that the livestock were personally raised by their owners for judging at a fair and therefore, the degree of direct contact was likely greater and the management conditions different than what would be typical in most commercial cattle operations. However, most of the cattle were raised in small pens of only a few cattle per pen on multiple rural properties.

**Figure 3:**
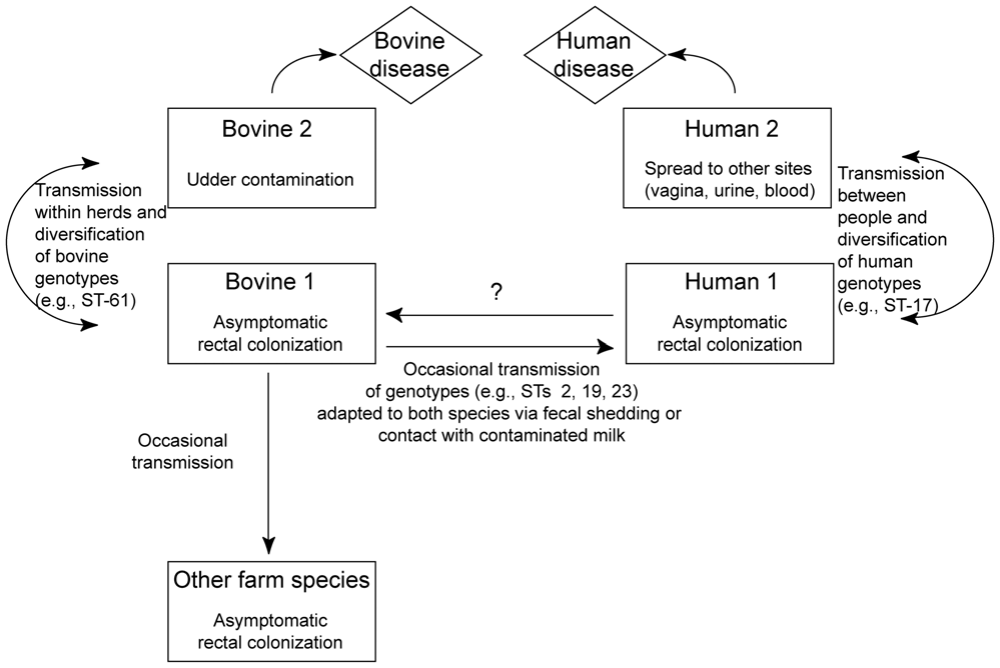
Hypothetical mode of group B *Streptococcus* (GBS) transmission between bovines and humans. It is possible that the diversification of GBS occurs independently in both humans and bovines, and only a subset of those strains can cause human and bovine disease. It is also possible that there is occasional transmission of GBS to other animal species (e.g, sheep, goats, etc.), particularly in a farm environment.

There are several explanations for the noted association between increasing cattle exposure and colonization. Because only stools were examined, it is plausible that milk was a source of transmission from bovines with culture-negative stools. Other anatomical sites, including the rectum or hide, also may have been colonized in bovines with negative stool specimens, which could partly explain the association with bovine exposure despite the low GBS prevalence. In addition, variation in GBS colonization density may be important for transmission. Even though density was not examined, GBS-positive humans and animals likely had dense colonization since stool versus rectal specimens were examined. Finally, it is likely that bovines have transient GBS colonization of the lower gastrointestinal tracts, which has been observed in humans [29], but has yet to be studied in bovines. Determining the frequency of GBS fecal shedding in livestock may be important for mastitis control programs and limiting transmission to humans and other farm animals (e.g., sheep). Based on these explanations, we hypothesize that there are multiple routes by which humans could acquire GBS from bovines, or vice versa (Figure 3).

Previously, it had been suggested that interspecies GBS transmission was unlikely because genotyping data demonstrated that human- and bovine-derived strains represent largely distinct populations [7], [8], [9], [10], [11], [12], [13]. While this is generally true, an examination of the data from prior MLST studies illustrates that several human-derived STs (e.g., ST-2, ST-17, ST-19, ST-23) are found in both species [10], [11], [13]. These STs represent close relatives or the most common STs in circulation among colonizing and invasive human strains in varying locations [10], [30], [31]. While STs 2, 19 and 23 have been observed in multiple bovine populations, all are significantly more common in humans [10], [11], [13]. Indeed, STs 1, 2, 19 and 23 occurred more frequently in colonized pregnant women than infected neonates [31], which may be attributable to variation in colonization abilities or transmissibility among genotypes in a range of species. In contrast, strains representing ST-17, a lineage suggested to be derived from a bovine ancestor [10], have only been found in two bovine milk samples from 1980 [11], but have been associated with invasive neonatal GBS disease in multiple populations [30], [31], [32], [33]. Whereas the pathogenic mechanism behind ST-17-mediated disease is not known, ST-17 strains and close relatives have distinct virulence gene profiles and may have diverged independently in human populations [15], [18]. Perhaps the same is true for GBS lineages specific to either bovines or humans, with independent divergence being influenced by high levels of recombination [18], [34].

Although the direction of transmission could not be established in this study, the identification of a shared GBS strain suggests that transmission occurred between the bovine and its' caretakers, or that all three were exposed to a common source. The latter explanation seems unlikely given the low GBS prevalence in bovines. It is also doubtful that contamination occurred given the careful specimen collection procedures. Data from a previous study [35] demonstrated that human- and bovine-derived GBS adhered to human epithelia similarly, but that bovine-derived strains adhered to vaginal epithelial and bovine milk cisternae cells at higher levels. Whereas this prior study did not examine differences between genotypes, the findings suggest that transmission from bovines to humans is more plausible than from humans to bovines [35]. This suggestion is supported by the observation that ST-1 and ST-19 strains solely colonized the throats of healthy subjects more than other anatomical sites, implying that the throat may be the site by which specific GBS clones enter the body [23]. In the present study, the strain shared between the bovine and its' caretakers was ST-1.

Together these findings suggest that GBS may be transmitted between bovines and humans in a farm environment and that increased cattle exposure is associated with colonization. Furthermore, several common GBS clones can colonize both humans and bovines and therefore, we hypothesize that these clones may be more readily transmitted between species (Figure 3). Such clones may also asymptomatically colonize livestock. Future longitudinal studies with more detailed sampling schemes are required to confirm these findings, identify risk factors, and estimate the frequency of GBS co-colonization in bovines and their caretakers. Such studies are necessary to fully understand the risk and frequency of interspecies GBS transmission.

## Methods

### Study Population

The U.S. 4-H organization is an agricultural program that introduces farming to children and young adults. As part of their experiential learning, members raise livestock for judging and auction at county fairs and therefore, they have considerable exposure to animals raised for exhibit. This high level of livestock exposure provides an opportunity to study the interspecies transmission of potential pathogens. We invited adolescents, parents and guardians enrolled in 4-H programs from three Michigan counties between June and August 2008 to participate in this cross-sectional study. The three adjacent central Michigan countries were selected because of their interest in animal husbandry projects involving porcines, bovines and equines.

### Ethics Statement

The Michigan State University and Michigan Department of Community Health Institutional Review Boards approved the study protocol. Written informed consent was obtained from all 180 participants or legal guardians for those younger than 18 years of age.

### Study Protocol

After providing informed consent, stools were self-collected from 1–2 family members and the animal to which they had the most frequent personal contact. The stool collection protocol stressed hand washing at two intermediary steps as well as after collection, and parental supervision was mandated for participants <18 years old. Each specimen was put into Cary Blair transport medium, which was placed into another seal-tight aluminum container to avoid cross-contamination. Several participants also reported close contact with a second farm animal and in these cases, an additional stool specimen was requested from this animal. The final analysis included all matched stools from bovines and their caretakers and the first 65 matched stools from other livestock/caretaker pairs. Each participant also completed a survey involving exposure levels to different animal species.

### GBS Characterization

Swabs from the Culture Swab Plus Collection System [Baltimore Biological Labs; Sparks, MD] were dipped into stools, inoculated in Todd-Hewitt broth containing colistin and nalidixic acid, and subcultured onto trypticase soy agar with 5% sheep blood; both were incubated overnight at 37°C in CO_2_. As described previously [4], the culture was tested for the group B antigen using the PathoDx kit [Remel Inc.; Lenexa, KS] and CAMP tests were performed on suspect colonies.

GBS isolates were characterized by capsule (*cps*) genotyping and multilocus sequence typing (MLST) of seven housekeeping genes as described elsewhere [36], [37]. Consensus sequences were trimmed in SeqMan (DNASTAR) and alleles and sequence types (STs) were assigned at http://pubmlst.org/sagalactiae/ [17]. A neighbor-joining tree was constructed in MEGA4 [38] using untransformed distances (*p* distance) and bootstrap confidence values (1000 replications). Susceptibilities to penicillin, ampicillin, erythromycin, azithromycin and clindamycin were determined by disk diffusion [39] as described previously [40].

To determine the similarity of matched GBS strains from animals and their caretakers, we used random amplified polymorphic DNA (RAPD) typing [9], which was previously shown to have a similar discriminatory index (>95%) as pulsed-field gel electrophoresis for GBS [41]. Three distinct RAPD primers were used and strains with identical capsular types, MLST, RAPD and antibiotic susceptibility profiles were considered to constitute probable transmission between linked family members and their animals.

### Data Analysis

The proportion of humans and animals with GBS colonization was calculated and the frequency of co-colonization among family members and their animals was determined. Crude odds ratios and 95% confidence intervals (CI) examined associations with GBS colonization; statistical significance was determined using the Likelihood Ratio Chi square (X^2^) or the Fisher's exact test. A logistic regression model was fit to determine the strongest predictors of colonization, and for single proportions, upper and lower confidence limits of the 95% CI were calculated with continuity correction [42].

## References

[1] Hansen SM, Uldbjerg N, Kilian M, Sorensen UB (2004). Dynamics of *Streptococcus agalactiae* colonization in women during and after pregnancy and in their infants.. J Clin Microbiol.

[2] Easmon CS, Hastings MJ, Deeley J, Bloxham B, Rivers RP (1983). The effect of intrapartum chemoprophylaxis on the vertical transmission of group B streptococci.. Br J Obstet Gynaecol.

[3] Manning SD, Neighbors K, Tallman PA, Gillespie B, Marrs CF (2004). Prevalence of group B *Streptococcus* colonization and potential for transmission by casual contact in healthy young men and women.. Clin Infect Dis.

[4] Manning SD, Schaeffer KE, Springman AC, Lehotzky E, Lewis MA (2008). Genetic diversity and antimicrobial resistance in group B *Streptococcus* colonizing young, nonpregnant women.. Clin Infect Dis.

[5] Edwards MS, Rench MA, Palazzi DL, Baker CJ (2005). Group B streptococcal colonization and serotype-specific immunity in healthy elderly persons.. Clin Infect Dis.

[6] Bruner DW, Tucker EW (1953). Methods for typing streptococci causing bovine mastitis.. Cornell Vet.

[7] Finch LA, Martin DR (1984). Human and bovine group B streptococci: two distinct populations.. J Appl Bacteriol.

[8] Mosabi JM, Arimi SM, Kang'ethe EK (1997). Isolation and characterization of group B streptococci from human and bovine sources within and around Nairobi.. Epidemiol Infect.

[9] Martinez G, Harel J, Higgins R, Lacouture S, Daignault D (2000). Characterization of *Streptococcus agalactiae* isolates of bovine and human origin by randomly amplified polymorphic DNA analysis.. J Clin Microbiol.

[10] Bisharat N, Crook DW, Leigh J, Harding RM, Ward PN (2004). Hyperinvasive neonatal group B *Streptococcus* has arisen from a bovine ancestor.. J Clin Microbiol.

[11] Bohnsack JF, Whiting AA, Martinez G, Jones N, Adderson EE (2004). Serotype III *Streptococcus agalactiae* from bovine milk and human neonatal infections.. Emerg Infect Dis.

[12] Sukhnanand S, Dogan B, Ayodele MO, Zadoks RN, Craver MP (2005). Molecular subtyping and characterization of bovine and human *Streptococcus agalactiae* isolates.. J Clin Microbiol.

[13] Oliveira IC, de Mattos MC, Pinto TA, Ferreira-Carvalho BT, Benchetrit LC (2006). Genetic relatedness between group B streptococci originating from bovine mastitis and a human group B *Streptococcus* type V cluster displaying an identical pulsed-field gel electrophoresis pattern.. Clin Microbiol Infect.

[14] Manning SD, Tallman P, Baker CJ, Gillespie B, Marrs CF (2002). Determinants of co-colonization with group B *Streptococcus* among heterosexual college couples.. Epidemiology.

[15] Brochet M, Couve E, Zouine M, Vallaeys T, Rusniok C (2006). Genomic diversity and evolution within the species *Streptococcus agalactiae*.. Microbes Infect.

[16] Keefe GP (1997). *Streptococcus agalactiae* mastitis: a review.. Can Vet J.

[17] Jolley KA, Chan M-S, Maiden MC (2004). mlstdbNet-distributed multi-locus sequence typing (MLST) database.. BMC Bioinformatics.

[18] Springman AC, Lacher DW, Wu G, Milton N, Whittam TS (2009). Selection, recombination and virulence gene diversity among group B streptococcal genotypes.. J Bacteriol.

[19] Bliss SJ, Manning SD, Tallman P, Baker CJ, Pearlman MD (2002). Group B *Streptococcus* colonization in male and nonpregnant female university students: a cross-sectional prevalence study.. Clin Infect Dis.

[20] Hammerschlag MR, Baker CJ, Alpert S, Kasper DL, Rosner I (1977). Colonization with group B streptococci in girls under 16 years of age.. Pediatrics.

[21] Islam AK, Thomas E (1980). Faecal carriage of group B streptococci.. J Clin Pathol.

[22] Persson KM, Bjerre B, Elfstrom L, Polberger S, Forsgren A (1986). Faecal carriage of group B streptococci.. Eur J Clin Microbiol.

[23] van der Mee-Marquet N, Fourny L, Arnault L, Domelier AS, Salloum M (2008). Molecular characterization of human-colonizing *Streptococcus agalactiae* strains isolated from throat, skin, anal margin, and genital body sites.. J Clin Microbiol.

[24] Sargeant JM, Scott HM, Leslie KE, Ireland MJ, Bashiri A (1998). Clinical mastitis in dairy cattle in Ontario: frequency of occurrence and bacteriological isolates.. Can Vet J.

[25] Pitkala A, Haveri M, Pyorala S, Myllys V, Honkanen-Buzalski T (2004). Bovine mastitis in Finland 2001–prevalence, distribution of bacteria, and antimicrobial resistance.. J Dairy Sci.

[26] Tenhagen BA, Hansen I, Reinecke A, Heuwieser W (2009). Prevalence of pathogens in milk samples of dairy cows with clinical mastitis and in heifers at first parturition.. J Dairy Res.

[27] Brglez I, Stropnik Z, Batis J (1986). Phagetypes of human and bovine *Streptococcus agalactiae* isolates in Slovenia.. Zentralbl Bakteriol Mikrobiol Hyg B.

[28] Jensen NE, Aarestrup FM (1996). Epidemiological aspects of group B streptococci of bovine and human origin.. Epidemiol Infect.

[29] Foxman B, Gillespie B, Manning SD, Howard LJ, Tallman P (2006). Incidence and duration of group B *Streptococcus* by serotype among male and female college students living in a single dormitory.. Am J Epidemiol.

[30] Jones N, Oliver KA, Barry J, Harding RM, Bisharat N (2006). Enhanced invasiveness of bovine-derived neonatal sequence type 17 group B *Streptococcus* is independent of capsular serotype.. Clin Infect Dis.

[31] Manning SD, Springman AC, Lehotzky E, Lewis MA, Whittam TS (2009). Multilocus sequence types associated with neonatal group B streptococcal sepsis and meningitis in Canada.. J Clin Microbiol.

[32] Luan SL, Granlund M, Sellin M, Lagergard T, Spratt BG (2005). Multilocus sequence typing of Swedish invasive group B *Streptococcus* isolates indicates a neonatally associated genetic lineage and capsule switching.. J Clin Microbiol.

[33] Lin FY, Whiting A, Adderson E, Takahashi S, Dunn DM (2006). Phylogenetic lineages of invasive and colonizing strains of serotype III group B streptococci from neonates: a multicenter prospective study.. J Clin Microbiol.

[34] Brochet M, Rusniok C, Couve E, Dramsi S, Poyart C (2008). Shaping a bacterial genome by large chromosomal replacements, the evolutionary history of *Streptococcus agalactiae*.. Proc Natl Acad Sci U S A.

[35] Kubin V, Haskova V, Jiraskova Z, Franek J (1983). Adherence of group B streptococci isolated from man and cattle to human and bovine epithelial cells.. Folia Microbiol (Praha).

[36] Manning SD, Lacher DW, Davies HD, Foxman B, Whittam TS (2005). DNA polymorphism and molecular subtyping of the capsular gene cluster of group B *Streptococcus*.. J Clin Microbiol.

[37] Manning SD, Lewis MA, Springman AC, Lehotzky E, Whittam TS (2008). Genotypic diversity and serotype distribution of group B *Streptococcus* isolated from women before and after childbirth.. Clin Infect Dis.

[38] Tamura K, Dudley J, Nei M, Kumar S (2007). MEGA4: Molecular Evolutionary Genetics Analysis (MEGA) software version 4.0.. Mol Biol Evol.

[39] Clinical and Laboratory Standards Institute (January 2007). Performance standards for antimicrobial susceptibility testing; seventeenth informational supplement..

[40] Manning SD, Pearlman MD, Tallman P, Pierson CL, Foxman B (2001). Frequency of antibiotic resistance among group B *Streptococcus* isolated from healthy college students.. Clin Infect Dis.

[41] Duarte RS, Miranda OP, Bellei BC, Brito MA, Teixeira LM (2004). Phenotypic and molecular characteristics of *Streptococcus agalactiae* isolates recovered from milk of dairy cows in Brazil.. J Clin Microbiol.

[42] Newcombe RG (1998). Two-sided confidence intervals for the single proportion: comparison of seven methods.. Stat Med.

